# Cell nuclei extraction from breast cancer histopathologyimages using colour, texture, scale and shape information

**DOI:** 10.1186/1746-1596-8-S1-S5

**Published:** 2013-09-30

**Authors:** Antoine Veillard, Maria S Kulikova, Daniel Racoceanu

**Affiliations:** 1 IPAL– UMI CNRS, 1 Fusionopolis Way, #21-01 Connexis, 138632 Singapore; 2 School of Computing, National University of Singapore, 117417 Singapore

## Background

Standard cancer diagnosis and prognosis procedures such as the Nottingham Grading System for breast cancer incorporate a criterion based on cell morphology known as cytonuclear atypia. Therefore, algorithms able to precisely extract the cell nuclei are a requirement in computer-aided diagnosis applications.

**Figure 1:**
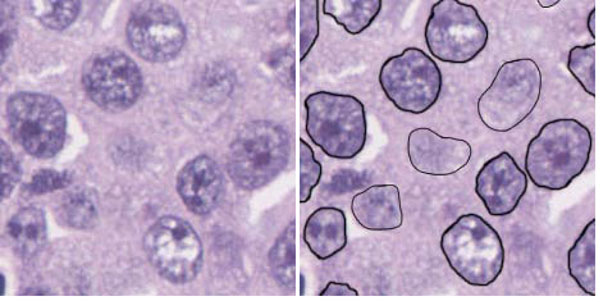
**Ground truth** (Left) magnified 250 × 250 region of a frame and (right) the same region with the nuclei delineated by a pathologist. Nuclei delineated with a thinner outline are hard to distinguish from the background. Dark and bright areas can indiscriminately occur inside and outside nuclei.

However, unlike other modalities such as needle aspiration biopsy images, H&E stained surgical breast cancer slides are a particularly challenging image modality due to the heterogeneity of both the objects and background, low object-background contrast and frequent overlaps as illustrated in Figure 1. As a consequence, existing extraction methods which are largely reliant on color intensities do not perform well on such images.

## Materials and methods

We propose a method based on the creation of a new image modality consisting in a grayscale map where the value of each pixel indicates its probability of belonging to a cell nuclei. This probability map is calculated from texture and scale information in addition to simple pixel color intensities. The resulting modality has a strong object-background contrast and evens out the irregularities within the nuclei or the background. The actual extraction is performed using an AC model with a nuclei shape prior included to deal with overlapping nuclei.

### Feature model

First, a color deconvolution [1] is applied in order to separate the immunohistochemical stains from which 3 grayscale images are produced: a haematoxilin image, an eosin image and a third residual component orthogonal in RGB space. Next, local features based on Laws’ texture measures [2] are computed for each pixel of the 3 obtained images. 5 different 1-dimensional convolution kernels (L5 = (*1*, *4*, *6*, *4*, *1*), *E_5_* = (*–1*, *–2*, *0*, *2*, *1*), *W_5_* = (*–1*, *2*, *0*, *–2*, *1*), *S_5_* = (*–1*, *0*, *2*, *0*, *–1*) and *R_5_* = (*1*, *–4*, *6*, *–4*, *1*)) are used to compute 25 different 5 × 5 kernels by convolving avertical 1-dimensional kernel with a horizontal one. The 5 × 5 kernels are applied at every pixel to extract 25 features which are then combined into 15 rotationally invariant features after normalizing by the output of the *L_5_^T^* × *L_5_* kernel and smoothing with a Gaussian kernel of standard deviation *σ* = *1.5* pixels.

The same process is repeated at 4 different scales after locally re-sampling the image using Lanczos-3 sinc kernels. Re-sampled images are locally computed around each pixel to allow the computation of the 15 texture features for the same pixel at different scales. Local texture features are computed at 1:1, 1:2, 1:4 and 1:8 scales for every pixel.

### Probability map

The resulting 180-dimensional feature vector *x* is used to compute the probability *p_n_*(*x*) of each pixel to belong to a cell nuclei. Let *μ_n_* (resp. *μ_b_*) be the mean of the feature vectors for the pixels belonging to the nuclei (resp. to the background). A class dependent LDA is performed in order to find two directions in the feature space, *w_n_* and *w_b_*, such that the projection of the classes on these directions has a maximum inter-class scatter over within-class scatter ratio. The estimated class probability associated with the feature vector x is then calculated from the linear scores *l_n_* = (*x – μ_n_*) *· w_n_* and *l_b_* = (*x – μ_b_*) *· w_b_* using the softmax function:

The resulting probability map exhibits strong contrast between the objects and the background. Moreover, nuclei and background appear more homogeneous than in the original image. A post processing step is also applied to fill small holes still remaining in nuclei (larger holes are not removed to prevent the unintended deletion of interstices between different nuclei).

### AC model including shape prior

The actual extraction of cell nuclei is performed from the probability map with an AC model with shape prior information. The total energy *E*(*γ*) associated to a contour γ is a weighted sum of an image term *E_i_*(*γ*) and a shape term *E_s_*(*γ*). The latter is itself the weighted sum of a smoothing term *E_sm_*(*γ*) and a shape prior term *E_sp_*(*γ*).

The shape prior term  allows to control the perturbations *δr*(*t*) of a contour around a circle at different frequencies *k* of the Fourier components by adjusting the coefficients *f_k_*. Detailed formulas and explanations for this and the other energy terms can be found in the work of Kulikova *et al. *[3]. The shape prior information allows to properly extract overlapping nuclei according to their expected shape without arbitrarily discarding the overlapping parts.

The detection of nuclei is performed by a marked point process model the details about which the interested reader can find in [4]. An empirical study in [5] shows that this particular combination of MPP and AC over-performs other state-of-the-art methods for nuclei detection and extraction.

## Results and discussion

The training set used for the LDA consists of 6 1024×1024 images where the nuclei have been manually delineated by a pathologist. Object and background parameters used in the AC model are also calculated from the training set. Weight parameters for the energy terms in the AC model are adjusted with a grid search. Images used for training are distinct from the images used for validation.

**Figure 2:**
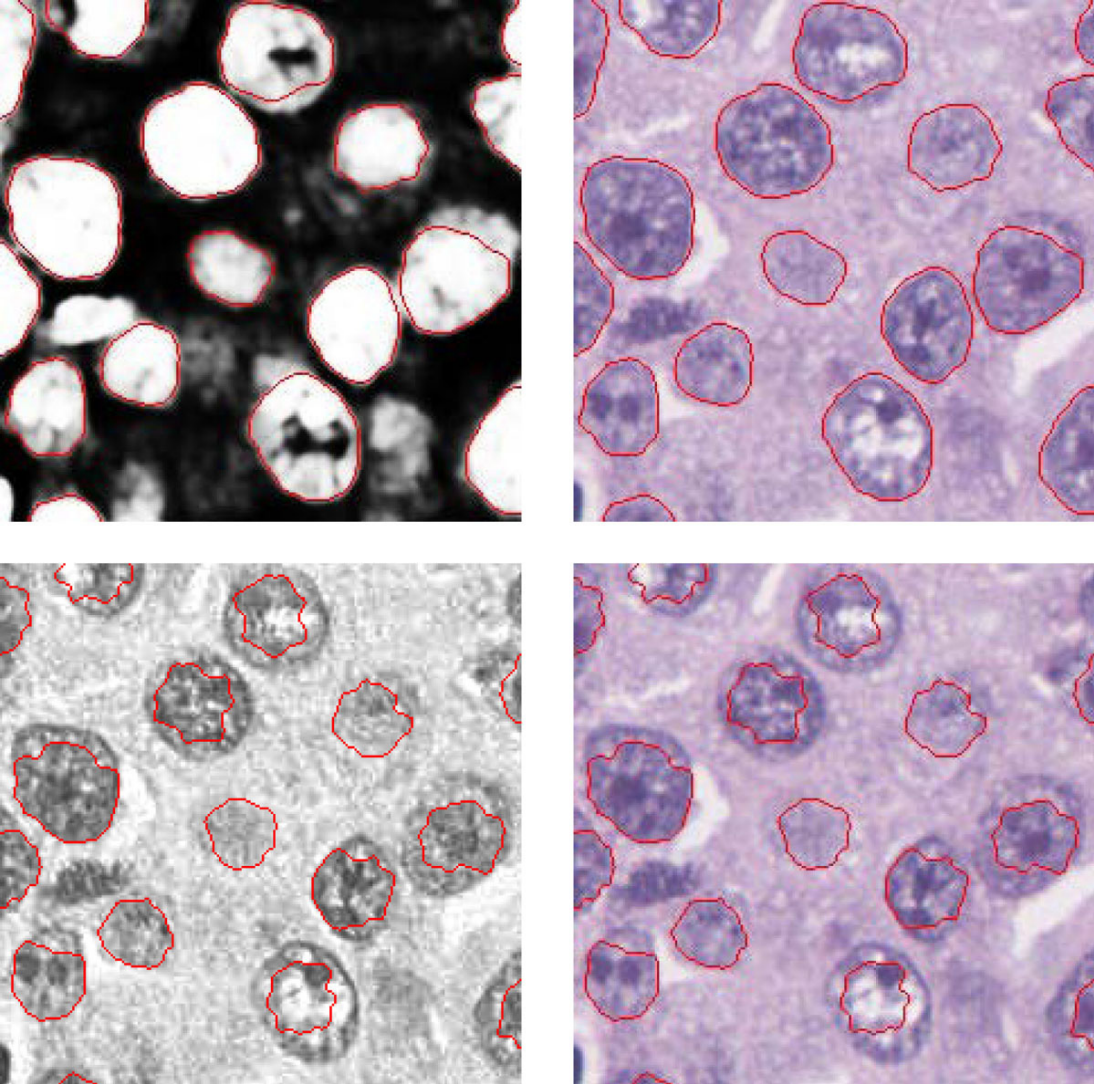
**Extraction results** The top row is obtained using the probability map and the bottom row is obtained using the haematoxilin channel after the image color deconvolution.

Figure 2 shows results obtained with the AC model applied to the probability map side-by-side with results obtained with the same AC model applied to the original image (in fact, the slightly better performing haematoxilin image from the color deconvolution was used instead of the red channel from the RGB image commonly used in other methods [6]). On the original image, the contours have a tendency to match irregularities within the cell nuclei rather than their actual boundaries. This problem is largely improved by using the probability map where the nuclei boundaries are much more salient and other irrelevant features are smoothed out.
